# On the Cure of the Elephantiasis

**Published:** 1793

**Authors:** At'har Ali' Khán

**Affiliations:** of Dehli.


XV. *On the Cure of the Elephantiafis. By* At'har Ali' Khán, *of Dehli.* Vide *Afiatick Refearches: or, Tranfactions of the Society inftituted in Bengal, for inquiring into the Hiftory and Antiquities, the Arts, Sciences, and Literature of Afia. Volume II.* 4to. Calcutta, 1790.

IN an introductory note to this paper, Sir William Jones, the learned prefident of the fociety, obferves, that the difeafe which is the fubject of it has been long known in Hinduftan, and that the writer of it, whofe father was phyficiain fician to Nádir Sháh, and accompanied him from Persia to Dehli, has assured him that it rages with virulence among the native inhabitants of Calcutta.

This disease, Sir William remarks, is the *judhám* of the Arabs, or *khórah* of the Indians. It is also, he adds, called in Arabia *dáül'ásad*, a name corresponding with the *leontiasis* of the Greeks, and supposed to have been given in allusion to the grim, distracted, and *lion-like* countenances of the miserable persons who are affected with it. The more common name of the distemper is *elephantiasis*, or, as Lucretius calls it, *elephas*, because it renders the skin, like that of an elephant, uneven and wrinkled, with many tubercles and furrows; but this complaint the learned president observes, must not be confounded with the *dáül'fil*, or swelled legs, described by the Arabian physicians, and very common in India. It has no fixed name in English, though Hillary, in his observations on the diseases of Barbadoes, calls it the *leprosy of the joints*, because it principally affects the extremities, which, in the last stage of the malady are distorted, and at length drop off: but since it is in truth a distemper corrupting the whole mass of blood, and therefore considered by

Paulus

Paulus Ægineta as an univerſal ulcer, it requires, Sir William thinks, a more general appellation, and may properly be named the *black leproſy*; which term is in fact adopted by Sauvages and Gorræus, in contradiſtinction to the white leproſy, or the *beres* of the Arabs, and *leuce* of the Greeks.

This diſeaſe, by whatever name we diſtinguiſh it, is peculiar to hot climates, and has rarely appeared in Europe. Lucretius ſuppoſes it confined to the banks of the Nile; and it has certainly been imported from Africa into the Weſt India iſlands by the black ſlaves.

With reſpect to the remedy recommended for the cure of this diſeaſe in Hinduſtan, the learned preſident obſerves, that whatever apprehenſions may be formed of future danger from the diſtant effect of arſenick, even though it ſhould eradicate a preſent malady, yet as no ſuch inconvenience has ariſen from the uſe of it in India, and as experience muſt ever prevail over theory, he cannot help wiſhing, that this ancient Hindu medicine may be fully tried under the inſpection of the European ſurgeons in that country. Should it, he adds, be thought that a mixture of ſulphur muſt render the poiſon leſs active, it may be adviſable, at firſt, to adminiſter nifter orpiment, inftead of the cryftalline arfenick.

We come now to the paper itfelf, which we fhall here give entire.

‘ God is the all-powerful healer.

‘ In the year of the Meffiah 1783, when the ‘ worthy and refpectable *Maulavi* MI'R MUHAMMED HUSAI'N, who excels in every ‘ branch of ufeful knowledge, accompanied Mr. ‘ Richard Johnfon from Lac'knau to Calcutta, ‘ he vifited the humble writer of this tract, ‘ who had long been attached to him with fin- ‘ cere affection, and in the courfe of their con- ‘ verfation, “ One of the fruits of my late ex- “ curfion, faid he, is a prefent for you, which “ fuits your profeffion, and will be generally “ ufeful to our fpecies: conceiving you to be “ worthy of it by reafon of your affiduity in “ medical inquiries, I have brought you a pre- “ fcription, the ingredients of which are eafily “ found, but not eafily equalled, as a powerful “ remedy againft all corruptions of the blood, “ the *judham*, and the *Perfian* fire, the remains “ of which are a fource of infinite maladies. “ It is an old fecret of the Hindu phyficians; “ who applied it alfo to the cure of cold and

“ moift

" moiſt diſtempers, as the palſy, diſtortions of

" the face, relaxation of the nerves, and ſimilar

" diſeaſes : its efficacy too has been proved by

" long experience, and this is the method of

" preparing it.

" Take of white arſenic, fine and freſh, one

" *tólá*; of picked black pepper ſix times as

" much : let both be well beaten at intervals

" for four days ſucceſſively in an iron mortar,

" and then reduced to an impalpable powder in

" one of ſtone, with a ſtone peſtle, and thus

" completely levigated, a little water being

" mixed with them. Make pills of them as

" large as tares, or ſmall pulſe, and keep them

" dry in a ſhady place *.

" One

* The following note to the above paſſage is by Sir William Jones : ' The loweſt weight in general uſe among the ' Hindus is the *reti*, called in Sanſcrit either *rettica* or *rac-* ' *tica*, indicating *redneſs*, and *criſhnalà* from *criſhna*, *black* ; ' it is the *red* and *black* ſeed of the *gunjà*-plant, which is a ' creeper of the ſame claſs and order at leaſt with the *glycyr-* ' *rhiza* ; but I take this from report, having never examined ' its bloſſoms. One *rattica* is ſaid to be of equal weight ' with three barley corns, or four grains of rice in the huſk ; ' and eight *reti* weights, uſed by jewellers, are equal to ' ſeven carats. I have weighed a number of the ſeeds in ' diamond ſcales, and find the average apothecary's weight ' of

"One of thoſe pills muſt be ſwallowed morn-

"ing and evening with ſome *betel*-leaf, or, in

"countries where *betel* is not at hand, with

"cold water: if the body be cleanſed from

"foulneſs and obſtructions by gentle cathar-

"tics and bleeding, before the medicine is

"adminiſtered, the remedy will be ſpeedier."

'The principal ingredient of this medicine

'is the arſenic, which the Arabs call *ſhucc*, the

'Perſians *mergi múſh*, or *mouſe-bane*, and the

'Indians *ſanc'hyá*; a mineral ſubſtance ponde-

'rous and *cryſtalline*; the orpiment, or yellow

'arſenic, is the weaker ſort. It is a deadly

'poiſon, and ſo ſubtil, that when mice are kil-

'led by it, the very ſmell of the dead will de-

'ſtroy the living of that ſpecies; after it has

'of one ſeed to be a grain and five ſixteenths. Now in the

'Hindu medical books ten of the *rattica* ſeeds are one

'*máſhaca*, and eight *máſhacás* make a *tólaca* or *tólà*; but in

'the law books of Bengal a *máſhaca* conſiſts of *ſixteenratticás*,

'and a *tólaca* of five *máſhà's*; and, according to ſome authori-

'ties, *five reti's* only go to one *máſhà*, *ſixteen* of which make

'a *tólaca*. We may obſerve, that the ſilver *reti* weights, uſed

'by the Goldſmiths at Banáres, are *twice* as heavy as the

'*ſeeds*; and thence it is that *eight reti's* are commonly ſaid

'to conſtitute one *máſhà*; that is, *eight* ſilver weights, or

'*ſixteen* ſeeds; eighty of which ſeeds, or 105 grains, con-

'ſtitute the quantity of arſenic in the Hindu preſcription.'

‘ been kept about ſeven years it loſes much of its force: its colour becomes turbid, and its weight is diminiſhed. This mineral is hot and dry in the fourth degree; it cauſes ſuppuration, diſſolves or unites according to the quantity given; and is very uſeful in cloſing the lips of wounds, when the pain is too intenſe to be borne. An unguent made of it, with oils of any ſort, is an effectual remedy for ſome cutaneous diſorders; and, mixed with roſe water, it is good for cold tumours and for the dropſy: but it muſt never be adminiſtered without the greateſt caution; for ſuch is its power, that the ſmalleſt quantity of it in powder, drawn, like alcohol, between the eyelaſhes, would, in a ſingle day, entirely corrode the coats and humours of the eye; and fourteen *reti's* of it would in the ſame time deſtroy life. The beſt antidote againſt its effects are the ſcrapings of leather reduced to aſhes. If the quantity of arſenic taken be accurately known, four times as much of theſe aſhes, mixed with water and drank by the patient, will ſheath and counteract the poiſon.

‘ The writer, conformably to the directions of his learned friend, prepared the medicine;

‘ and,

'and, in the ſame year, gave it to numbers

'who were reduced by the diſeaſes above-men-

'tioned to the point of death. God is his wit-

'neſs, that they grew better from day to day,

'were at laſt completely cured, and are now

'living (except one or two, who died of other

'diſorders) to atteſt the truth of this aſſertion.

'One of his firſt patients was a *Pârſi*, named

'*Menúchehr*, who had came from Surat to this

'city, and had fixed his abode near the writer's

'houſe: he was ſo cruelly afflicted with a

'confirmed lues, here called *the Perſian fire*,

'that his hands and feet were entirely ulcerated

'and almoſt corroded, ſo that he became an

'object of diſguſt and abhorrence. This man

'conſulted the writer on his caſe, the ſtate of

'which he diſcloſed without reſerve. Some

'blood was taken from him the ſame day, and

'a cathartic adminiſtered on the next. On

'the third day he began to take the arſenic

'pills, and, by the bleſſing of God, the viru-

'lence of his diſorder abated by degrees, until

'ſigns of returning health appeared; in a fort-

'night his recovery was complete, and he was

'bathed, according to the practice of our phy-

'ſicians: he ſeemed to have no virus left in his

'blood,

'blood, and none has been ſince perceived by

'him.

'But the power of this medicine has chiefly 'been tried in the cure of the *juzám*, as the 'word is pronounced in India; a diſorder in'fecting the whole maſs of blood, and thence 'called by ſome *fiſádi khún*. The former name 'is derived from an Arabic root ſignifying, in 'general, *amputation*, *maiming*, *exciſion*, and par'ticularly the *truncation or eroſion of the fingers*, 'which happens in the laſt ſtage of the diſeaſe. 'It is extremely contagious, and for that rea'ſon, the prophet ſaid: *Ferrú mina'lmejdhúmi* '*camá teferrú mina'l áſad*, or, "Flee from a 'perſon afflicted with the judhám, as you would 'flee from a lion." The author of the *bahhru'l*-'*jawahir*, or *ſea of pearls*, ranks it as an infec'tious malady with the meaſles, the ſmall pox, 'and the plague. It is alſo hereditary, and, in 'that reſpect, claſſed by medical writers, with 'the gout, the conſumption, and the white 'leproſy.

'A common cauſe of this diſtemper is the 'unwholeſome diet of the natives, many of 'whom are accuſtomed, after eating a quantity 'of fiſh, to ſwallow copious draughts of milk,

'which

'which fail not to cauſe an accumulation of yel-

'low and black bile, which mingles itſelf with

'the blood, and corrupts it: but it has other

'cauſes; for a *bráhmen*, who had never taſted

'*fiſh* in his life, applied lately to the compoſer

'of this eſſay, and appeared in the higheſt de-

'gree affected by a corruption of blood, which

'he might have inherited, or acquired by other

'means. Thoſe, whoſe religion permits them

'to eat *beef*, are often expoſed to the danger of

'heating their blood intenſely, through the

'knavery of the butchers in the *bázár*, who

'fatten their calves with *baláwer*; and thoſe,

'who are ſo ill-adviſed as to take provocatives,

'a folly extremely common in India, at firſt

'are inſenſible of the miſchief, but, as ſoon as

'the increaſed moiſture is diſperſed, find their

'whole maſs of blood inflamed, and, as it were,

'aduſt; whence ariſes the diſorder of which

'we are now treating. The *Perſian* (or vene-

'real) fire generally ends in this malady; as

'one *Dévi Praſád*, lately in the ſervice of Mr.

'Vanſittart, and ſome others, have convinced

'me by an unreſerved account of their ſeveral

'caſes.

'It may here be worth while to report a re-
'markable caſe, which was related to me by a

 man,

'man, who had been afflicted with the *juzám* 'near four years; before which time he had 'been disordered with the *Persian* fire, and, 'having closed an ulcer by means of a strong 'healing plaister, was attacked by a violent 'pain in his joints. On this he applied to a '*cabirája*, or Hindu physician, who gave him 'some pills, with a positive assurance, that the 'use of them would remove his pain in a few 'days; and in a few days it was, in fact, wholly removed: but, a very short time after, the 'symptoms of the *juzam* appeared, which continually increased to such a degree, that his 'fingers and toes were on the point of dropping off. It was afterwards discovered, that 'the pills which he had taken, were made of 'cinnabar, a common preparation of the Hindus; the heat of which had first stirred the 'humours, which, on stopping the external 'discharge, had fallen on the joints, and there 'had occasioned a quantity of adu st bile to mix 'itself with the blood, and infect the whole 'mass.

'Of this dreadful complain t, however caused, the first symptoms are a numbness and 'redness of the whole body, and principally 'of the face; an impeded hoarse voice, thin hair,

‘ hair, and even baldneſs; offenſive perſpira-

‘ tion and breath, and whitlows on the nails.

‘ The cure is beſt begun with copious bleed-

‘ ing, and cooling drink, ſuch as a decoction

‘ of the *nilúfer*, or *nymphæa*, and of violets,

‘ with ſome doſes of manna: after which

‘ ſtronger cathartics muſt be adminiſtered. But

‘ no remedy has proved ſo efficacious as the

‘ pills compoſed of arſenic and pepper: one

‘ inſtance of their effects may here be mention-

‘ ed, and many more may be added, if re-

‘ quired.

‘ In the month of February, in the year juſt

‘ mentioned, one *Shaikh Ramazáni*, who was

‘ then an upper ſervant to the board of revenue,

‘ had ſo corrupt a maſs of blood, that a black

‘ leproſy of his joints was approaching, and

‘ moſt of his limbs began to be ulcerated: in

‘ this condition he applied to the writer, and

‘ requeſted immediate aſſiſtance. Though the

‘ diſordered ſtate of his blood was evident on

‘ inſpection, and required no particular decla-

‘ ration of it, yet many queſtions were put to

‘ him, and it was clear from his anſwers, that

‘ he had a confirmed *juzám*: he then loſt a

‘ great deal of blood, and, after due prepara-

‘ tion, took the arſenic pills. After the firſt

'week his malady ſeemed alleviated; in the 'ſecond it was conſiderably diminiſhed; and, 'in the third, ſo entirely removed, that the pa- 'tient went into the bath of health, as a token 'that he no longer needed a phyſician.'

